# Neosomes of tungid fleas on wild and domestic animals

**DOI:** 10.1007/s00436-014-4081-8

**Published:** 2014-08-21

**Authors:** Pedro Marcos Linardi, Daniel Moreira de Avelar

**Affiliations:** 1 Departamento de Parasitologia, Instituto de Ciências Biológicas da Universidade Federal de Minas Gerais, Caixa Postal 486, Avenida Antônio Carlos, 6627, Campus UFMG, Belo Horizonte, Minas Gerais 31270-901 Brazil; 2 Laboratório de Pesquisas Clínicas, Centro de Pesquisas René Rachou, Fundação Oswaldo Cruz, Belo Horizonte, Minas Gerais Brazil

**Keywords:** Neosome, Tungid fleas, Sand fleas, Wild and domestic animals, Siphonaptera

## Abstract

*Tunga* is the most specialized genus among the Siphonaptera because adult females penetrate into the skin of their hosts and, after mating and fertilization, undergo hypertrophy, forming an enlarged structure known as the neosome. In humans and other warm-blooded animals, neosomes cause tungiasis, which arises due to the action of opportunistic agents. Although its effects on humans and domestic animals are well described in the literature, little is known about the impact of tungiasis on wild animals. This review focuses on the morphology, taxonomy, geographical distribution, hosts, prevalence, sites of attachment, and impact of tungid neosomes on wild and domestic animals. Because neosomes are the most characteristic form of the genus *Tunga* and also the form most frequently found in hosts, they are here differentiated and illustrated to aid in the identification of the 13 currently known species. Perspectives for future studies regarding the possibility of discovering other sand flea species, adaptation to new hosts, and the transfer of tungids between hosts in natural and modified habitats are also presented.

**Electronic supplementary material:**

The online version of this article (doi:10.1007/s00436-014-4081-8) contains supplementary material, which is available to authorized users.

## Introduction

Adult fleas (Siphonaptera) are obligate hematophagous ectoparasites that infest humans and wild and domestic animals. There are approximately 3,000 species and subspecies of fleas included in 238 genera and 15 families worldwide (Lewis 1998). Tungidae is the most specialized family in that the females of the genera *Tunga* Jarocki (Tunginae) penetrate the skin of their hosts (Hopkins and Rothschild 1953; Linardi and Guimarães 2000), and after mating, the gravid females undergo hypertrophy, becoming neosomes (Audy et al. 1972) in spite of the genus *Neotunga* Smit of the family Pulicidae also present penetrating females. Another genus, *Hectopsylla* Frauenfeld (Hectopsyllinae), includes species that are also considered neosomatic, but the females are semipenetrating and only attach their mouthparts to the hosts.

According to Audy et al. (1972), neosomes are organisms that are altered by the formation of a new external morphological structure and the secretion of new cuticle, accompanied by significant enlargement during an active process of metamorphosis. Although neosomy exists in other Arthropoda, in fleas, the process occurs in approximately 90 sessile or semisessile species (Rothschild 1992), primarily in the families Vermipsyllidae and Tungidae. Neosomatic vermipsyllids, also called alakurt fleas, include two species—*Dorcadia ioffi* Smit and *Vermipsylla alakurt* Schimkewitsch—that parasitize ungulates, particularly domestic sheep, horses, and yaks in Central Asia, but their females do not burrow beneath the skin as do tungids, which are endoparasitic.

In tungids, neosomes are the most frequently observed form in hosts. Because neosomes involute with the death of the parasite after oviposition (Lavoipierre et al. 1979), specific identification can be difficult because certain characteristics cannot be observed in the most commonly dissected specimens. Later, the neosomes can be absorbed or sloughed from the host epidermis by tissue repair mechanisms (Eisele et al. 2003; Lavoipierre et al. 1979).

Whereas *Neotunga* is composed of only one valid species, *Neotunga euloidea* Smit, which parasitizes African pangolins (Lewis 1998), the genus *Tunga* includes 13 species of sand fleas (De Avelar et al. 2013). Two *Tunga* species are found in China and Japan, and the other 11 occur in the New World tropics. One of these, *Tunga penetrans* (L., 1758), also occurs in Sub-Saharan Africa (Beaucournu et al. 2012b; De Avelar et al. 2012; Lewis 1998; Linardi and Guimarães 2000).

However, because there is little knowledge of this group, some misidentifications have been recorded, especially given that many specimens collected from parasitological investigations or veterinary surveys were named according to their hosts. This circumstance is compounded by the fact that, with the exception of De Avelar et al. (2012), taxonomic keys rarely contain data on neosomes.

Tungiasis causes serious ectoparasitosis, and harmful infections and their effects on humans are well documented, especially in Heukelbach (2005, 2006) and Karunamoorthi (2013). With respect to domestic animals, a review can be found in Pampiglione et al. (2009). However, little is known about the impact of tungiasis on wild animals. The present article provides a selective review of the morphology, taxonomy, geographical distribution, hosts, prevalence, preferred sites of attachment, and impact of neosomes on wild and domestic animals. The paper also presents perspectives for future research regarding the possibility of discovering other sand flea species, adaptation to new hosts, and the interchange of tungids between hosts from natural and modified habitats.

## Morphology and taxonomy

Currently, the genus *Tunga* includes the following species: *T. penetrans* (L., 1758); *Tunga caecata* (Enderlein, 1901); *Tunga caecigena* Jordan & Rothschild, 1921; *Tunga travassosi* Pinto & Dreyfus, 1927; *Tunga bondari* Wagner, 1932; *Tunga terasma* Jordan, 1937; *Tunga callida* Li & Chin, 1957; *Tunga libis* Smit, 1962; *Tunga monositus* Barnes & Radovsky, 1969; *Tunga trimamillata* Pampiglione et al., 2002; *Tunga* *bossii* De Avelar et al., 2012; *Tunga bonneti* Beaucournu & González-Acuña, 2012; and *Tunga hexalobulata* De Avelar et al., 2013. *Tunga penetrans* is the most well-known species, having been described in the eighteenth century and referred to in the literature as the sand flea, sandflöh, puce de sable, chigoe, jigger, chigger, chique, nigua, and bicho-de-pé, among other names. In Peru alone, when searching for the evidence of tungiasis in pre-Hispanic America, Maco et al. (2011) used 35 different local names for *T. penetrans*.

Eight of the 13 species of the genus *Tunga* have known males: *T. penetrans*, *T. terasma*, *T. caecigena*, *T. callida*, *T. libis*, *T. monositus*, *T. trimamillata*, and *T. bonneti*. Four species—*T. travassosi*, *T. bondari*, *T.* *bossii*, and *T. hexalobulata*—are known only by their neosomes. Only two species—*T. penetrans* and *T. monositus*—have immature forms that have been described. The species *T. libis*, *T. bondari*, *T. bossi*, and *T. hexalobulata* are known only through a small number of specimens.

Neosomes vary in form and size, but the enlargement of the abdomen generally occurs between abdominal segments II and III, as seen in *T. caecigena* (Jordan 1962), *T. monositus* (Barnes and Radovsky 1969), and *T. penetrans* (Eisele et al. 2003). The posterior part has the form of a caudal disk-like or conical prominence that bears respiratory, anal, and genital apertures and is exposed through an opening in the host’s skin (Audy et al. 1972). In the dorsal view, the caudal disk is sclerotized, resembling a crater. Data regarding the morphology of neosomes are presented in Table 1. For the first time, the neosome of *T. bondari* is illustrated, although a brief description was included in De Avelar (2010). Neosomes of *T. travassosi* have been described with the head and thorax evaginated in relation to the abdomen (Pinto and Dreyfus 1927). However, after the observation of specimens deposited in the scientific collection of the Museum of Zoology of the University of São Paulo, Brazil, and the Department of Parasitology of the Federal University of Minas Gerais, Brazil, it was verified that such structures are, in fact, invaginated, and they can be seen only after dissection of the neosomes.

**Table 1:** Morphology and morphometry of the neosomes of *Tunga* species

*Tunga* species	Neosomes			
	Shape	Measurements (mm) (length × width × height)	Head and thorax in relation to the abdomen (lateral view)	Caudal disk (segments IV–X)
*T. penetrans*	Globular without lobes	6 × 5 × 4	Evaginated	Flattened, wider than long
*T. caecata*	Globular without lobes	7 × 6 × 6	Invaginated	Conical, almost as wide as long
*T. travassosi*	Globular without lobes	13 × 8 × 10	Invaginated	Conical, as wide as long
*T. terasma*	Subcylindrical with four lateral prominent lobes	10 × 9 × 13	Evaginated	Cylindrical, longer than wide
*T. bondari*	Mushroom-shaped with a stem, as that generated by a nuclear explosion	6 × 6 × 5	Evaginated	Cylindrical, longer than wide
*T. caecigena*	Elliptical with four lobes: dorsal and ventral portions of similar dilatation	7–10 × 5 × 6	Not visible in profile	Cylindrical, longer than wide
*T. callida*	Spherical with four lobes: dorsal portion more swollen than the ventral portion	4.5 × 4.5 × 4.5	Not visible in profile	Cylindrical, as long as wide
*T. libis*	Vertically elliptical and without lobes	higher than long	Not visible in profile	–
*T. monositus*	Bell-shaped with 8 lobes, arranged as 4 large outer lobes and 4 small inner lobes	6 × 5.4 × 4.5	Evaginated but not visible in profile	Flattened, wider than long
*T. trimamillata*	Globular with 3 lobes located anteriorly	12 × 5 × 5	Evaginated but not visible in profile	Conical, wider than long
*T. bossii*	Globular without lobes	9 × 8 × 7	Invaginated	Flattened, wider than long
*T. bonneti*	Horizontally elliptical with rugby ball shape	10 × 6	Invaginated	–
*T. hexalobulata*	Spherical with six lobes located anteriorly, pearl-white colored, slightly compressed in anterior direction	4 × 4 × 4	Evaginated but not visible in profile	Conical, wider than long

**Fig. 1:**
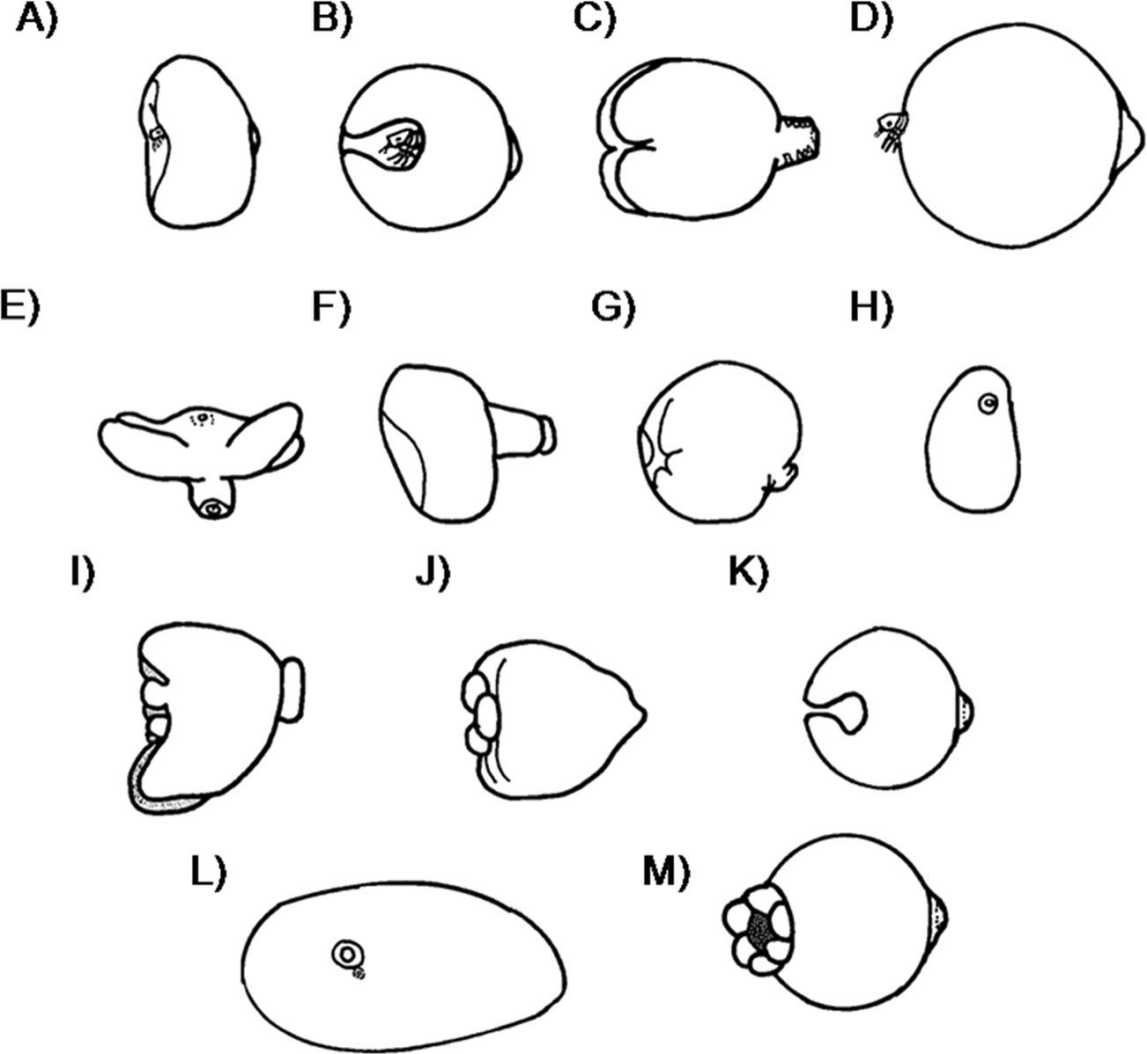
Shape of gravid females of the species of *Tunga*: **A** *T. penetrans*; **B** *T. caecata*; **C** *T. caecigena*; **D** *T. travassosi*; **E** *T. terasma*; **F** *T. bondari*; **G** *T. callida*; **H** *T. libis*; **I** *T. monositus*; **J** *T. trimamillata*; **K** *T. bossii*; **L** *T. bonneti*; **M** *T. hexalobulata*

**Fig. 2:**
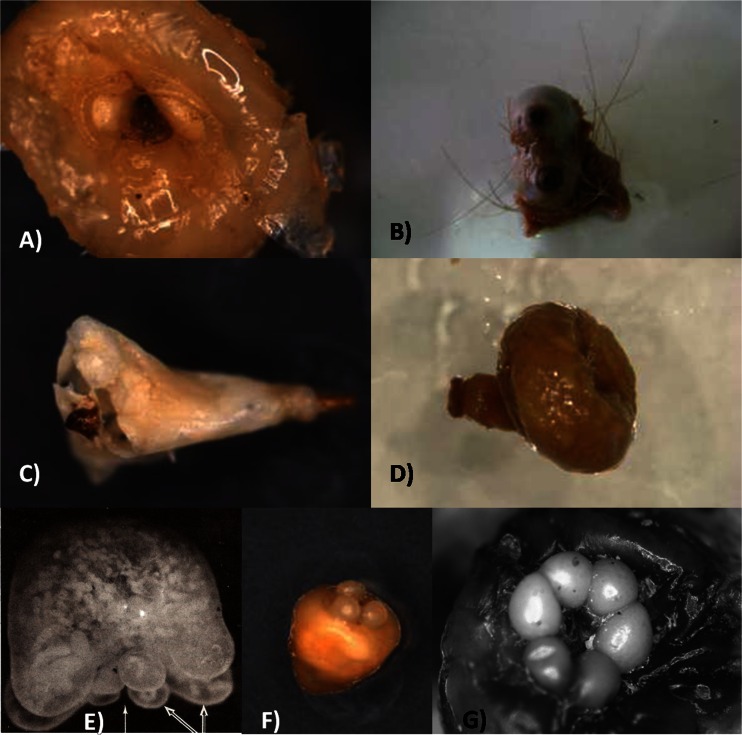
Neosomes of some species of *Tunga* after removal from their hosts: **A** *T. penetrans*; **B** *T. travassosi*; **C** *T. terasma*; **D** *T. bondari*; **E** *T. monositus* (after Lavoipierre et al. 1979); **F** *T. trimamillata*; **G** *T. hexalobulata*

Figure 1 shows the shape of the gravid females of *Tunga* species found both in wild and domestic animals. Figure 1l is a dorsal view of *T. bonneti*, whereas in the other panels, the neosomes are shown in a lateral view. Figure 2 shows neosomes after dissection from their respective hosts. A neosome of the semisessile flea *Hectopsylla pulex* (Haller, 1880) is included for comparison.

## Geographical distribution

Species of *Tunga* have been found from 34° 41′ N to 33° 29′ S and from 38° 30′ W to 135° 30′ E. According to Barnes and Radovsky (1969), the center of distribution and the apparent origin of the genus *Tunga* are in the Neotropical region. Indeed, of the 13 described species, nine are restricted to South America, and another, *T. penetrans*, exhibits a wide Neotropical distribution, as it is now permanently established in much of tropical Africa (Lewis 1998). In spite of occasional records of *Tunga* fleas on humans in the USA (Bell et al. 1979; Brothers and Heckmann 1975; Goldman 1976; Reiss 1966; Sanusi et al. 1989), Italy (Veraldi et al. 1996; Veraldi and Valsecchi 2007), and New Zealand (Pilgrim 1993), sand fleas do not appear to have established themselves in these countries.

De Avelar (2010) described the presence of *T. penetrans* in the following countries: Argentina, Brazil, Colombia, Chile, Ecuador, Guyana, French Guiana, Paraguay, Peru, Suriname, Trinidad, Uruguay, Venezuela, and the Caribbean Islands in Latin America and South Africa, Angola, Botswana, Cameroon, Congo, Ivory Coast, Eritrea, Gabon, Ghana, Guinea, Liberia, Libya, Madagascar, Mali, Mauritania, Mozambique, Namibia, Niger, Nigeria, Kenya, Central African Republic, São Tomé, Senegal, Sierra Leone, Somalia, Sudan, Tanzania, Tunisia, Uganda, Zaire, Zambia, Zanzibar, and Zimbabwe in Sub-Saharan Africa. According to Pampiglione et al. (2009), tungiasis has been reported in approximately 70 nations.

Except for *T. penetrans*, Table 2 presents the geographical distribution by country and respective state or province where the species have been recorded as well as the ranges of distribution and altitude. Eight of the 13 species occur in Brazil, six of which are endemic in that country. Although the extent of the distribution depends upon the number of localities recorded for the species, and although *T.* *bossii* and *T. hexalobulata* have been found only in the localities in which they were described, the ranges of the distribution of each species, which vary from 494 to 5,465 km, may also indicate the level of dispersion of each species. Similarly, certain species, such as *T. trimamillata*, *T. libis*, and *T. bonneti*, which are found in the Andean regions, can also occur at high altitudes. Except for new Brazilian occurrences (Table 3), all of the records of other localities of capture were detailed and presented by Beaucournu et al. (2012b) along with their geographical coordinates.

**Table 2:** Geographical distribution of species of the genus *Tunga*

Species	Countries: states or provinces	Limits of distribution			References
		Localities (from/to)	Extension (km)	Altitude (m)	
*T. caecata*	Brazil: Minas Gerais (MG), Paraná (PA), São Paulo (SP)	Caratinga MG/Curitiba PA	965	533 to 1,141	Linardi and Guimarães (2000)
*T. travassosi*	Brazil: SP, MG	Sorocaba SP/Belo Vale MG	494	566 to 800	Linardi and Guimarães (2000)
*T. terasma*	Brazil: Goiás (GO), MG, SP, Espírito Santo (ES), Mato Grosso do Sul (MS)	Anápolis GO/São Paulo SP	965	98 to 1,141	Linardi and Guimarães (2000); Antunes et al. (2006)
*T. bondari*	Brazil: Bahia (BA), SP, MG	Salvador BA/Franca SP	1,268	0 to 1,141	Hopkins and Rothschild (1953); Linardi and Guimarães (2000)
*T. caecigena*	China: Chekiang (CH), Fukien (FU), Kiangsu (KI), Shanghai (SH), Szechuan (SZ), Soochow	Nishinomyia HY/Futsing FU	839	9 to 229	Jordan (1962); Beaucournu et al. (2012b)
	Japan: Honshu (HO), Osaka (OS), Hyogo (HY)				
*T. callida*	China: Yunnan (YU)	–	–	–	Li and Chin (1957)
*T. libis*	Ecuador: Chimborazo (CM)	Riobamba CM/Reserva Nacional Las Chinchilas	3,418	0 to 2,200	Smit (1962); Beaucournu et al. (2012b)
	Chile: Chañaral (CA), Choapa (CH)				
*T. monositus*	Mexico: Baja California (BC), San Martin Island (SM)	Washington County UT/San Quintin Bay (BC)	841	0	Barnes and Radovsky (1969); Hastriter (1997)
	USA: Utah (UT)				
*T. trimamillata*	Ecuador: Azuai (AZ), Loja (LO), Santa Isabel, Catacocha, Machala Peru: Piura (PI) Brazil: SP, MG	Sujo PI/Felixlândia MG	5,465	29 to 2,709	Fioravanti et al. (2006); Ribeiro et al. (2007); Linardi et al. (2013); Luchetti et al. (2005a)
*T. bossii*	Brazil: Rio de Janeiro (RJ)	Itatiaia National Park RJ	–	2,359	De Avelar et al. (2012)
*T. bonneti*	Chile: Atacama (AT), Santiago (SA), Limarí (LI), Huasco (HU), Antofagasta (AN), El Loa (EL)	Quebrada de Inca EL/Dunas Las Cruces SA	1,405	0 to 3,794	Beaucournu et al. (2012a)
*T. hexalobulata*	Brazil: MG	Funilândia MG	–	692	De Avelar et al. (2013)

**Table 3:** New occurrences of species of *Tunga*

Species of *Tunga*	New occurrences	
	Localities/states	References
*T. caecata*	Caratinga/MG: 19° 47′ S/44° 52′ W, 586 m Nova Lima/MG: 20° 07′ S/43° 51′ W, 817 m São Paulo/SP: 23° 32′ S/46° 38′ W, 761 m São João da Boa Vista/SP: 21° 58′ S/46° 48′ W, 774 m	De Avelar (2010); Linardi and Guimarães (2000); De Moraes et al. (2003)
*T. travassosi*	Belo Vale/MG: 20° 24′ S/44° 01′ W, 800 m	Linardi (unpublished)
*T. terasma*	Alegre/ES: 20° 45′ S/41° 29′ W, 150 m Pantanal da Nhecolândia: MS: 18° 59′ S/56° 39′ W, 98 m	Antunes et al. (2006); Medri (2008)
*T. trimamillata*	Barretos/SP: 20° 33′ S/48° 34′ W, 556 m Rio Novo/MG: 21° 28′ S/43° 07′ W, 416 m Felixlândia/MG: 18° 44′ S/44° 52′ W, 652 m	Vaz and Rocha (1946); Rodrigues and Daemon (unpublished work); Ribeiro et al. (2007); Linardi et al. (2013)
*T. hexalobulata*	Funilândia/MG: 19° 22′ S/44° 03′ W, 692 m	De Avelar et al. (2013)

It is important to stress that all new occurrences are recorded from Brazil.

*MG* Minas Gerais, *SP* São Paulo, *ES* Espírito Santo, *MS* Mato Grosso do Sul

## Hosts

Because the degree of specificity among flea/host species is variable, both fleas and hosts can be classified by the pattern of their relationships to separate natural and casual associations (Holland 1964; Krasnov 2008; Marshall 1981; Wenzell and Tipton 1966). In these circumstances, hosts can be considered as true, primary, accidental, or secondary for a given species of flea. True hosts, also called normal, essential, or primary hosts, are those that provide favorable conditions under which a flea species can reproduce indefinitely. However, according to Holland (1964), the primary host is derived from ancient or even original associations. Accidental hosts are those that are due purely to chance and may also include erroneous records arising from a mistaken host or flea identification; however, as noted by Sakaguti and Jameson (1962), some cases considered to be accidental hosts might, in fact, be alternative true hosts.

A secondary host is an intermediate category for those considered neither a true nor an accidental host. According to Holland (1964), this category is transitional for some animals because a secondary host may eventually become a true host. According to Marshall (1981), from the ectoparasitological viewpoint and depending upon the species number of their true hosts, fleas can be regarded as one of the following: monoxenous (only one host), oligoxenous (two or more host species in the same genus), pleioxenous (two or more host genera in the same family), or polyxenous (several hosts in multiple families).

Among the 13 known species of *Tunga*, *T. penetrans* is the most promiscuous, having been found on hosts belonging to eight different orders of mammals, including Cingulata, Pilosa, Artiodactyla, Perissodactyla, Carnivora, Rodentia, Primates, and Proboscidea; in total, *T. penetrans* has been found on 27 genera of wild and domestic animals (De Avelar 2010) in addition to two occurrences that have only recently been recorded (Frank et al. 2012; Widmer and Azevedo 2012). However, in spite of the broad spectrum and category of hosts, the pig is considered the most important animal reservoir for *T. penetrans* (Pampiglione et al. 1998; Ugbomoiko et al. 2008). Humans and dogs might be considered secondary hosts or even true or essential hosts undergoing the process of adaptation. The parasitism of *T. penetrans* on elephants (Ruthe 1961), gorillas (Ewing and Fox 1943), and monkeys (Fitzsimmons 1966) should be seen as accidental. However, because the identification of this species is not always performed by experts and is sometimes based on the sole criterion of “penetrating fleas,” some misidentifications may have occurred. In fact, records of *T. penetrans* on bats (Blanchard 1890) may be attributed to the sessile flea *H. pulex*. Similarly, the infestation of this species on *Gallus gallus* cited by Macchiavello (1948) might have been confused with the stick-tight flea *Echidnophaga gallinacea* (Westwood, 1875), and the parasitism on the passerine *Volatinia jacarina* reported in Lima and Hathaway (1946), if not accidental, must be attributed to *Hectopsylla psittaci* Frauenfeld, 1860. Similarly, the occurrence of *T. bondari* on *Cariama cristata*, cited by Hopkins and Rothschild (1953), is likely an accidental finding.

Concerning domestic animals, until now, only three species of sand fleas have been found as ectoparasites: *T. penetrans* on the pig, cow, dog, cat, and horse; *T. trimamillata* on the cow, goat, sheep, and pig; and *T. hexalobulata*, which was recently found on cattle. Only *T. penetrans* and *T. trimamillata* parasitize humans. The other 10 species exclusively infest wild animals, regardless of the number of host species. These species are listed in Table 4 and are based on Beaucournu et al. (2012b), Cunha (1914), Hopkins and Rothschild (1953), Johnson (1957), Lima and Hathaway (1946), Linardi and Guimarães (2000), Pinto (1930), and several studies included in De Avelar (2010). Host nomenclature follows Wilson and Reeder (2005).

**Table 4:** Hosts species for *Tunga* species

Species of *Tunga*	Type: number of true hosts	Species of mammal hosts
*T. penetrans*	Polyxenous	Artiodactyla: *Bos taurus*, *Sus scrofa*, *Capra hircus*, *Ovis aries*, *Pecari tajacu*, *Lama glama*, *Vicugna vicugna*, *Potamochoerus porcus* Carnivora: *Canis familiaris*, *Felis catus*, *Panthera onca* Cingulata: *Dasypus novencinctus*, *D. hybridus*, *Chaetophractus villosus* Perissodactyla: *Tapirus terrestris*, *Equus cabalus*, *Equus* sp. Pilosa: *Tamandua tetradactyla*, *Myrmecophaga tridactyla* Primates: *Homo sapiens*, *Gorilla gorilla*, *Papio* sp. Proboscidea: *Loxodonta africana* Rodentia: *Cuniculus paca*, *Dasyprocta punctata*, *Mus musculus*, *M. musculoides*, *Rattus rattus*, *Rattus norvegicus*, *Cavia porcellus*, *Cavia aperea*, *Myoprocta acouchy*, *Hystrix* sp.
*T. caecata*	Polyxenous	Rodentia: *M. musculus*, *R. rattus*, *R. norvegicus*, *Akodon cursor*, *Necromys pixuna*, *Nectomys squamipes*, *Oligoryzomys nigripes*, *Oxymycterus* sp., *Rhypidomys mastacalis*
*T. travassosi*	Monoxenous	Cingulata: *Dasypus novemcinctus*
*T. terasma*	Pleioxenous	Cingulata: *Cabassous unicinctus*, *Dasypus novemcinctus*, *Euphractus sexcinctus*, *Priodontes maximus*
*T. bondari*	Monoxenous	Pilosa: *Tamandua tetradactyla*
*T. caecigena*	Polyxenous	Rodentia: *R. rattus*, *R. norvegicus*, *M. musculus*, *Mus bactrianus* Insectivora: *Suncus murinus*
*T. callida*	Pleioxenous	Rodentia: *Rattus* sp., *R. rattus*, *R. norvegicus*, *Apodemus chevrieri*, *M. bactrianus*, *Eothenomys custos*
*T. libis*	Pleioxenous	Rodentia: *Akodon mollis*, *Phyllotis andium*, *Phyllotis darwini*
*T. monositus*	Pleioxenous	Rodentia: *Peromyscus maniculatus*, *P. eremicus*, *P. crinitus*, *Neotoma lepida*, *Neotoma* sp.
*T. trimamillata*	Polyxenous	Artiodactyla: *Bos taurus*, *Sus scrofa*, *Capra hircus*, *Ovis aries* Primates: *Homo sapiens* Rodentia: *Hydrochoerus hydrochoerus*
*T. bossii*	Monoxenous	Rodentia: *Delomys dorsalis*
*T. bonneti*	Oligoxenous	Rodentia: *Phyllotis darwini*, *P. xanthopygus*
*T. hexalobulata*	Monoxenous	Artiodactyla: *Bos indicus*
*Tunga* sp. (*caecata* group)	–	Rodentia: *Akodon montensis*, *Delomys sublineatus*, *Oligoryzomys nigripes* Didelphimorphia: *Monodelphis americana*

Another category of host is experimental animals, such as *Mus musculus* (Lavoipierre et al. 1979) and Wistar rats (an albino strain of *Rattus norvegicus*), which are used to establish the life cycle of *T. penetrans* in the laboratory (Feldmeier et al. 2007; Nagy et al. 2007), or dogs, which are used to test the efficacy of drugs against tungiasis (Klimpel et al. 2005).

According to Smit (1962), the genus *Tunga* comprises two species groups that are separated by morphological characteristics and host affinities: *caecata* and *penetrans*. In the *caecata* group, which is considered the most primitive, the following species are currently included, all of which exclusively parasitize rodents: *T. caecata*, *T. caecigena*, *T. callida*, *T. libis*, *T. monositus*, *T. bossii*, and *T. bonneti*. The finding of *T. caecigena* on *Suncus murinus* (Insectivora) might be accidental. The *penetrans* group is the evolutionarily advanced, as it is composed of *T. travassosi*, *T. bondari*, *T. terasma*, *T. trimamillata*, *T. hexalobulata*, and obviously *T. penetrans*, with the first three species associated primarily with edentates.

However, regardless of Smit’s proposal and according to Traub (1980), fleas generally parasitize the hosts with which they evolved. Thus, primitive hosts tend to have primitive fleas, whereas the most advanced mammals are associated with the most evolutionarily recent fleas. Thus, the primitive hosts of sand fleas might have been animals such as the Edentata, which are devoid of means to remove the neosomes (incisor teeth, nails) inserted in the infested sites (feet, belly, skin of abdomen). It is also assumed that tungids and their primitive edentate hosts occurred in Pangaea, and later, with the fragmentation of the continental blocks, both were isolated in South America, with the fleas then undergoing adaptive radiation and infesting new hosts (Traub 1985). After the disappearance of the primitive hosts in certain regions, such as the Nearctic and Palearctic, rodents became the primary reservoirs (Mascarenhas 2002).

A recent study based on the molecular phylogeny of fleas indicated that the basal position in the cladogram and the association of several species of the *penetrans* group with basal mammals (sloths and armadillos) suggest that the origin and diversification of Siphonaptera coincide with the diversification of basal mammals (Whiting et al. 2008). Possibly, mammals such as sloths (Pilosa) and armadillos (Cingulata) are the primary hosts, with other mammals (pigs, dogs, cats, rats, etc.) corresponding to secondary associations (Whiting et al. 2008). Wild pigs are also speculated to have been the primitive hosts, despite the meager records thus far reported.

It is known that domestic animals came to Brazil soon after its colonization by Europeans. At that time, wild animals such as the wild pig and the capybara had already been domesticated by indigenous natives. Thus, the extent to which wild and domestic pigs in the same areas and locations might have interchanged their ectoparasites is still unknown. In fact, in Angola, *T. penetrans* was found infesting the wild suid *Potamochoerus porcus* (Ribeiro 1974).

## Infestation

Flea infestations on hosts are usually defined by their mean abundance and prevalence. From an epidemiological point of view, mean abundance and prevalence have different meanings. Abundance, formerly known as the rate of infestation (or infection) or the index of parasitism (Marshall 1981), is a parameter that has been little used, mainly because the parasite load and intensity of infestation have been considered, incorrectly, to be synonymous by some ectoparasitologists. Mean abundance, however, is a parameter that can be employed as an indicator of the state of health of the host. Thus, high abundance might be related to the inability of the host to oppose the action of the parasite by means of its immune system and/or its behavior (for example, by grooming), as suggested by Stanko et al. (2002). In this respect, the effect of the age and sex of the host on parasite abundance requires further investigation because the defensive capacity of mammals may well increase or decrease over time and ectoparasite grooming might be more prevalent in one of the sexes. Multiple infestations of a single host by ectoparasites of different taxonomic groups may also influence the mean abundance of fleas, as these infestations are mediated by several types of ecological associations (intra- and interspecific competition, predation, mutualism, etc.). Furthermore, an increase in the mean abundance of fleas might reflect the increasing mortality of a host following infection by a pathogen.

Although most adult fleas spend a part of their time in the nest and certain species are almost exclusively nest inhabitants as adults, mammal fleas are commonly found on their hosts’ bodies, often in considerable numbers. In neosomatic fleas, the largest abundance recorded on a single individual was 7,000 alakurt *Dorcadia* from a sheep (Ioff 1950 in Marshall 1981). In tungids, 31 *T. caecigena* were counted on a *Rattus* species (Jordan 1962). In *T. travassosi*, tens of neosomes can be found in the belly of its host, *Dasypus novemcinctus*. Regarding *T. penetrans*, approximately 80 lesions were found on dogs, 50 on cats, 16 on rats, and 199 on humans from Fortaleza, Brazil (Heukelbach et al. 2004a), whereas in a community in rural Nigeria, Ugbomoiko et al. (2008) observed the following maximum numbers of lesions: 184 on pigs, 21 on dogs, and 13 on *Rattus rattus*. In a study of flea infestations of *Panthera onca*, Widmer and Azevedo (2012) recorded more than 20 lesions on a young female.

Prevalence is also related to the propagation of ectoparasites on their respective hosts. Thus, a high prevalence might be the result of a microenvironmental overlap among hosts that, when associated with environmental factors, would favor the development of immature stages. In this context, prevalence would be related to spatial factors, including the territoriality and dispersion of the host. Given the vectorial capacity of fleas, prevalence potentially represents a tool with which to measure the dissemination of pathogens.

Infestations vary according to host age, sex, size, behavior, host mobility, habitat, and climate (Marshall 1981). The preference for host females can be related to hormonal cycles, as observed between *Spilopsyllus cuniculi* and *Oryctolagus cuniculus* and between *Cediopsylla simplex* and *Sylvilagus* spp. Preferences for male hosts, as observed in rats, may exist because males have larger home ranges, are generally larger than females, and exhibit territorial behavior. Other sex and age preferences result from grooming because males are more efficient groomers than females and adults groom more than young individuals. Regarding environmental factors, Linardi and Krasnov (2013) observed that in three hosts (*Monodelphis domestica*, *Necromys lasiurus*, and *Oligoryzomys eliurus*) collected at different localities across Brazil, the mean flea abundance significantly increased with an increase in the mean annual air temperature and the proximity to the equator. For both *M. domestica* and *N. lasiurus*, abundance also decreased with altitude.

However, interpretations based on comparisons of these parameters should be made with caution when considering data from different regions that were not obtained in the same period. Moreover, the mode of capture, i.e., the number of trappings/unit time, is rarely described and varies among different studies; the baits used are not always the most suitable and can vary in both quality and quantity; hosts may belong to different taxonomic groups with different habits and habitats; and the sites of parasitism are not always the same on different hosts. Perhaps the most important factor in quantifying parameters, however, relates to the timing of fleas leaving the host. Fleas leave those hosts that have been confined in traps the longest and abandon the carcass completely after death (Marshall 1981; Pollitzer 1954). Consequently, more accurate data would be obtained as soon as possible after capture and, if possible, while still in the field. However, this situation does not apply to sand fleas in which gravid females remain attached to the hosts even after their death.

In spite of these variables, some available figures for prevalence on certain hosts at different locations and times are shown separately for *T. penetrans* (Table 5) and other sand fleas (Table 6). As shown in Table 5, with the exception of the data from Rodrigues et al. (2008), the prevalence of tungiasis in stray dogs is similar in poor communities and shanty towns: 45.5 to 60.9 %.

**Table 5:** Prevalence of infestation by *T. penetrans* on hosts in different locations

Location, state, country	Hosts				References
	Species	Examined (no.)	Infested (no.)	Prevalence (%)	
Coqueiros do Sul, RS, Brazil	Pigs	72	64	88.9	Pedroso-de-Paiva et al. (1997)
São Tomé Island, Africa		100	28	28.0	Pampiglione et al. (1998)
Araruama, RJ, Brazil		12	2	16.6	Carvalho et al. (2003)
Rural zone, Nigeria		31	17	54.8	Ugbomoiko et al. (2008)
Curitiba, PR, Brazil	Dogs	7.811	58	0.7	Vernalha et al. (1984)
Araruama, RJ, Brazil		123	75	60.9	Carvalho et al. (2003)
Fortaleza, CE, Brazil		150	76	50.7	Heukelbach et al. (2004a)
Rural zone, Nigeria		11	5	45.5	Ugbomoiko et al. (2008)
Juiz de Fora, MG, Brazil		101	2	2.0	Rodrigues et al. (2008)
Manaus, AM, Brazil		42	20	47.6	Corrêa et al. (2012)
Araruama, RJ, Brazil	Cats	24	2	8.3	Carvalho et al. (2003)
Fortaleza, CE, Brazil		158	72	45.6	Heukelbach et al. (2004a)
Fortaleza, CE, Brazil	Rats	34	14	41.2	Heukelbach et al. (2004a)
Rural zone, Nigeria	*R. rattus*	17	5	29.4	Ugbomoiko et al. (2008)
Rural zone, Nigeria	*M. minutoides*	13	2	15.4	Ugbomoiko et al. (2008)
Buenos Aires, BA, Argentina	*C. villosus*	4	1	25.0	Ezquiaga et al. (2008)
Buenos Aires, BA, Argentina	*D. hybridus*	13	1	7.7	Ezquiaga et al. (2008)
Uberlândia, MG, Brazil	*M. tridactyla*	3	2	66.7	Frank et al. (2012)
Pantanal region, MS, Brazil	*P. onca*	12	12	100	Widmer and Azevedo (2012)

**Table 6:** Prevalence of infestation by some sand fleas on the respective hosts

*Tunga* species	Hosts				Location	References
	Species	Collected (no.)	Infested (no.)	Prevalence (%)		
*T. caecata*	*R. norvegicus*	824	82	9.9	São Paulo, SP, Brazil	Meira (1934)
	*R. rattus*	445	13	2.9		
	*M. musculus*	135	2	1.5		
*T. caecigena*	*R. rattus*	250	68	27.2	Soochow, China	Wu (1930) in Jordan (1962)
	*R. norvegicus*					
*T. terasma*	*D. novemcinctus*	34	4	11.7	Alegre, ES, Brazil	Antunes et al. (2006)
	*E. sexcinctus*	31	1	3.2	Pantanal da Nhecolândia, MS, Brazil	Medri (2008)
*T. monositus*	*P. eremicus*					
	*P. crinitis*	21	6	28.6	Washington County, UT, USA	Hastriter (1997)
	*N. lepida*					
*T. trimamillata*	*Bos taurus*	170	130	76.4	Felixlândia, MG, Brazil	Ribeiro et al. (2007)
*T. trimamillata* or *T. penetrans*	*Bos taurus*	550	375	68.2	Jataí, GO, Brazil	Da Silva et al. (2001)

Considering that the bovine tungiasis previously attributed to *T. penetrans* in Barretos, São Paulo State, Brazil (Vaz and Rocha 1946), and Felixlandia, Minas Gerais State, Brazil (Ribeiro et al. 2007), was in fact caused by *T. trimamillata* (Linardi et al. 2013), it is likely that records pertaining to Jataí, Goiás State (Da Silva et al. 2001), and Santa Fé do Sul, São Paulo State (Moraes et al. 1992), are also of *T. trimamillata*.

## Sites of Attachment

Tungid fleas show consistent preferences for attachment sites. The semipenetrating flea *H. pulex* prefers bats’ heads, especially body regions with sparse hairs, such as the ears (Esbérard 2001), although these fleas are also found on the tragus, shoulder blade and tibia, anus, wings, axilla, mouth, and dactylopatagium (Luz et al. 2009). On pangolins, the pulicid *N. euloidea* is usually attached to the soft skin of the ventral region.

Among sand fleas, *T. penetrans* occurs commonly between the toes and periungueal regions when parasitizing humans (Eisele et al. 2003), justifying the vernacular name *bicho-de-pé* in Brazil (Cunha 1914; Linardi and Guimarães 2000). This species also can be found on other sites, such as the hands, soles, elbows, neck, anus, gluteal area, genital region, buttocks, heels, groin, face, etc. (Bezerra 1994; Heukelbach et al. 2002). Some authors (Cardoso 1981; Clyti et al. 2003) have also reported multiple sites spread over a large part of the body on a single person.

On pigs, *T. penetrans* occurs particularly on the feet, snout, and scrotum (Cooper 1976) but also on the mammary glands (Pedroso-de-Paiva et al. 1997) and in the calcaneal region (Pampiglione et al. 2009). On both dogs and cats, neosomes can be seen around the claws, on the pads and, especially, on the muzzle (Heukelbach et al. 2004a). Female dogs can also exhibit lesions in the nipples (Klimpel et al. 2005). On Brazilian wild mammals, Frank et al. (2012) reported lesions on the feet of the giant anteater *Myrmecophaga tridactyla*, and on the jaguar (*P. onca*), Widmer and Azevedo (2012) observed neosomes confined to the animals’ paws.

When infesting bovines, goats, and sheep, neosomes of the species *T. trimamillata* are found along the coronary band, in the coronary and digital cushions, and sometimes on the sole of the hoof (Pampiglione et al. 2009). In cattle, this species can also be observed on the edge of the nail wall development, that is, at the level of the perioptic tafe, as well as on the perianal area, on the udder of cows and prepuce of bulls (Vaz and Rocha 1946). On pigs, neosomes of this species also tend to be localized on the calcaneal region and scrotum in males and the udder in females (Pampiglione et al. 2009).

**Fig. 3:**
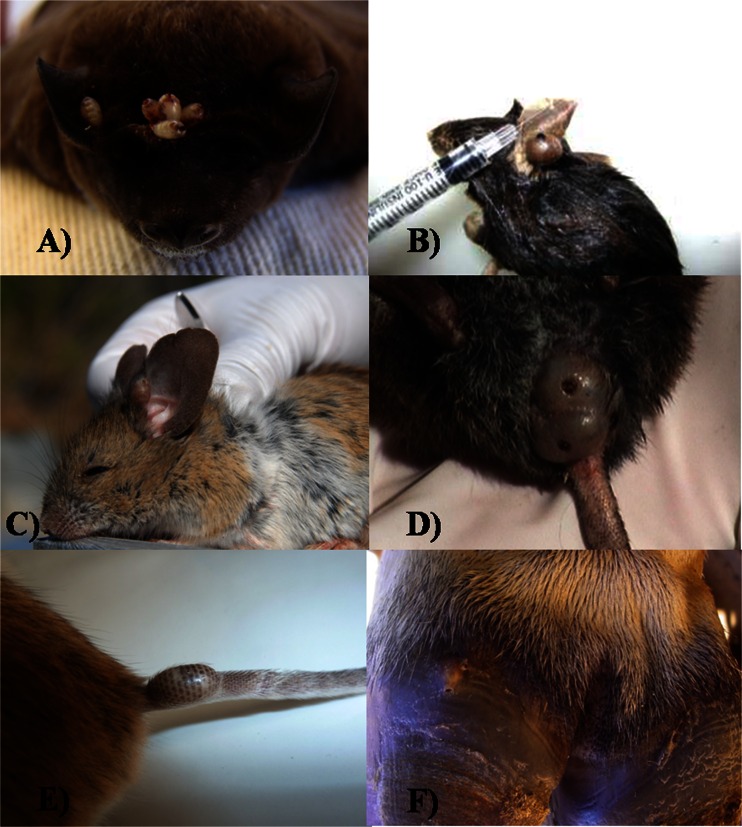
Sites of attachment of some neosomes on their hosts: **A** *H. pulex* on the bat’s head *Molossus* sp. (courtesy of Júlia Lins Luz); **B** *T. caecata* on the ear of *Oryzomys nigripes*; **C** *T. libis* on the ear of *Phyllotis darwini* (after Beaucournu et al. 2012a); **D** *T. bossii* on the tail of *Delomys dorsalis*; **E** *T. bonneti* on the tail of *P. darwini* (after Beaucournu et al. 2012a); **F** *T. trimamillata* on the hoof of Holstein-Zebu cow (courtesy of Elias Jorge Facury Filho)

Other species of *Tunga* exhibit the following preferential attachment sites on their respective hosts: *T. caecata*, upper surface of rat ears (Jordan 1962); *T. travassosi*, dermis of the ventral abdominal region of edentates (Lima 1943; Pinto 1930); *T. terasma*, ventral abdomen and toes of edentates (Antunes et al. 2006); *T. bondari*, ventral abdomen of edentates (Wagner 1932); *T. caecigena*, at the edge of the pinna but also on the dorsal surface of rat ears and, in one case (only on a specimen of *R. rattus*), at the base of the tail (Jordan 1962; Yang 1955); *T. callida*, rear end of the body, especially around the anus of rats (Li and Chin 1957); *T. libis*, ears of rodents (Beaucournu et al. 2012a); *T. monositus*, basal portion of the upper surface of the pinna of rodents (Barnes and Radovsky 1969); *T. bossii*, base of the tail of wild rodents (De Avelar et al. 2012); *T. bonneti*, parallel to the great axis of the tail of rodents (Beaucournu et al. 2012a); and *T. hexalobulata*, coronary band of cattle (De Avelar et al. 2013). Figure 3 shows sites of attachment for neosomes of some tungids on their hosts, including *H. pulex*.

Considering the data recorded thus far, the most frequent attachment sites are the feet, ventral abdominal, ears, and tails of hosts. The choice of these respective sites depends upon the following factors: (i) regions that regularly contact the soil, such as the feet in humans and domestic and wild animals or the ventral abdominal region in both domestic and wild mammals; (ii) areas from which the hosts have the greatest difficulty dislodging the parasites by grooming or eating, such as the ears and tail in rats or the ventral abdominal region in edentates, which are devoid of incisor teeth and nails; and (iii) the structure of the hair and thickness of the coat related to microclimates such as temperature and skin structure, as in bats and pangolins (Marshall 1981). According to Nagy et al. (2007), older cats and dogs have fewer neosomes than young individuals because of the thicker skin on their paws. Because species of *Tunga* have a reduced pleural arch (Traub 1972) and, consequently, are unable to jump very high, the lesions produced by *T. penetrans* are more concentrated or confined to the feet of animals, as reported by Klimpel et al. (2005).

## Impact on the hosts

The impact of tungid fleas depends on the sites of attachment on their hosts. The effects of parasitism are well known for *T. penetrans*, as it is the best known and disseminated species in the genus, infesting humans and domestic and wild animals.

In humans, the symptoms, direct impact, and complications have been well documented. Briefly, intense pain and itching are perceived to be the most irritating symptoms (Feldemeier et al. 2004), with most infestations occurring on the feet and provoking deformation of the digits and the loss of toenails. In some cases, severe inflammation and deep skin fissures prevent individuals from walking normally (Ariza et al. 2007; Heukelbach et al. 2007; Hoeppli 1963; Matias 1989). In the Kasulu district, western Tanzania, Mazigo et al. (2012) observed the following symptoms in individuals examined for tungiasis: itching (68.3 %), pain (38.6 %), ulcers (30.1 %), difficulty walking (22.1 %), and loss of toenails (21.3 %).

Superinfected lesions, primarily caused by *Staphylococcus aureus* and Gram-negative bacteria, lead to the formation of pustules, suppuration, and ulcers that may also be a port of entry for *Clostridium tetani* (Greco et al. 2001; Obengui 1989; Soria and Capri 1953; Tonge 1989). Other pathogenic microorganisms isolated from superinfected lesions include *Streptococcus pyogenes*, Enterobacteriaceae, *Bacillus* spp., *Enterococcus faecalis*, *Pseudomonas* spp., and various other anaerobic bacteria (Feldmeier et al. 2002; Heukelbach et al. 2007). Symptoms and signs used to determine the severity of acute tungiasis have been reported by Kehr et al. (2007).

Other complications include cases of gangrene, lymphangitis, lymphadenitis, and sepsis (Veraldi and Schianchi 1999). Brumpt (1949) reported a strain of *Yersinia pestis* isolated from *T. penetrans* in the Congo. Using molecular techniques, *Wolbachia* was also identified in a neosomic female *T. penetrans* from Ghana (Fischer et al. 2002), Brazil (Heukelbach et al. 2004b) as well as in swine from localities in Ecuador and in humans from different localities in Burundi, Kenya, and Zaire (Luchetti et al. 2004; 2005b).

Despite the effects of tungiasis in humans, very little is known of its parasitism on animals, especially wild mammals. In pigs, the infestation causes walking difficulties due to the presence of the parasite in the hoofs. When the lesions are located in the teats, they can cause agalactia and mastitis in lactating sows due to obstruction of the galactophorous channel, blocking milk production and subsequent involution of the mammary glands and causing starvation of the piglets (Pedroso-de-Paiva et al. 1997; Verhulst 1976).

Infested dogs change their position constantly, lick their pads, and are reluctant to stand up (Heukelbach et al. 2004a). In severe cases, dogs are unable to walk and may even die (Wolffhügel 1910), presumably due to the superinfection of lesions, leading to septicemia. Loss of digits can also be observed in rat tungiasis with severe inflammation (Heukelbach et al. 2004a).

Infestation on the feet of giant anteaters provokes inflammation that prevents the animals from breaking up termitaria with their forefeet, thus preventing them from accessing their main food. According to Frank et al. (2012), tungiasis most likely prevents or at least hinders this natural behavior, and as a consequence, the anteaters avoid common food sources in preference for those that are less suitable but more accessible.

*T. trimamillata* is the second species for which the effects on its hosts are known. In humans, local inhabitants of the Andean regions that have been co-infested by *T. penetrans* and *T. trimamillata* report that the infestation caused by the latter is more painful (Pampiglione et al. 2009). In bovines, infestation causes deformation of the nails. Some animals become completely crippled, refusing to walk or even unable to stand. When such injuries occur in a breeding animal, its inability to sustain its own weight can be compounded by pregnancy. The process is often complicated by secondary infection with ulceration on the upper edge of the nails as a consequence of the rupture of the neosomes caused by excoriation. In some cases, flies deposit their eggs in the ulcers that are so formed, increasing the destruction of the living tissue (Vaz and Rocha 1946). *Wolbachia* was also found in samples of *T. trimamillata* collected from goats and cattle in Santa Isabel, Ecuador, and identified through PCR amplification and sequencing of the bacterial ribosomal 16S and *fts*Z genes, with prevalences of 40 and 11 %, respectively (Luchetti et al. 2004, 2005b).

Little is known about the impact of other species of *Tunga* on wild animals. In contrast to *T. penetrans*, in which both males and females are blood feeding (Geigy and Herbig 1949; Witt et al. 2004), the larvae and adult males of *T. monositus* do not feed (Lavoipierre et al. 1979). According to these authors, after becoming attached to rodent skin, adult females of *T. monositus* feed principally off of fluid exudate and, for a few days, off of neutrophils, fibroblasts, collagen, and macrophages. Blood feeding commences from the 14th day.

Neosomes of sand fleas can reach a diameter of 1 cm or more (Table 1). *T. penetrans* may remain attached to the host for a period of more than 5 weeks (Eisele et al. 2003), whereas *T. monositus* lies embedded in the skin of the ear pinna for 2 or 3 months (Lavoipierre et al. 1979). As in other ectoparasitoses, the lesions may affect weight gain and milk production, and as in humans and domestic animals, the lesions might also become infected and be a port of entry for various pathogens. Considering the abundance of neosomes, their attachment sites and affected organs, as well as the lifespan of the embedded sand fleas, what is the real impact of these infestations on wild animals? Armadillos are insectivores that use the toes of the forefeet for digging into galleries to feed chiefly on ants, termites, and other small invertebrates. They have also been observed to roll about on ant hills to dislodge and consume the resident ants. How do we evaluate the action of tens of neosomes of *T. travassosi* on the toes or the 30 cm ventral surface of armadillos? Or similarly, more than 30 neosomes of *T. caecigena* over the ears or the tail of a rat that measure, respectively, 24 and 180 mm? It is important to stress that in rats, the pinna can be rotated to catch the slightest sound from almost any direction. This ability is useful for an animal whose activity is primarily confined to darkness. Rats also control their body temperature through their tails by dilating or constricting their tail blood vessels and use their tails for balance (Yulong et al. 1995). In the same manner, to what extent will the presence of neosomes of *H. pulex* on the ears of bats inhibit their ability to navigate, considering that the ears act as biosonar-receiving antennas?

## Perspectives

Among the 13 species currently included in the genus *Tunga*, more than 30 % have been described in the last 12 years; thus, the chances of discovering new species are high. Some samples of *T. penetrans* have displayed slight variations when collected from different hosts in Fortaleza, Brazil, which may indicate the existence of different strains or races or the beginning of such a formation (Nagy et al. 2007). Recently, De Avelar and Linardi (2010) showed that the multiple displacement amplification (MDA) technique enables the amplification of the genomic DNA of siphonapterids that have been preserved for long periods, with the successful amplification of one neosome of *T. bondari* that was collected in 1909. This technique may be a valuable tool for molecular studies involving samples of sand fleas that are preserved in scientific collections.

The Neotropical region stands out as the most promising region in which new taxa might be found, as it contains a diversity of biomes and at least 40 dispersion centers, 13 of which are located in Brazil (Müller 1972). Furthermore, Brazil is considered a hotspot of global biodiversity (Mittermeier et al. 1998; Myers et al. 2000), as it occupies a vast area and contains approximately 13 % of the world’s mammal fauna (Reis et al. 2006) that are recognized as valid species (Wilson and Reeder 2005). Rodents, representing approximately 36 % of the Brazilian mammals, deserve particular attention because in this country, rodents host five different *Tunga* species that are included in the *caecata* group. Additionally, considering that three species of *Tunga* that belong to the *penetrans* group are primarily associated with edentates (Hopkins and Rothschild 1953; Lima and Hathaway 1946; Linardi and Guimarães 2000) and that only seven of the 14 species of Brazilian Myrmecophagidae (Pilosa) and Dasypodidae (Cingulata) (Reis et al. 2006) have been recorded as hosts for these sand fleas, both armadillos and anteaters are taxa that should be further examined for occurrences of tungids because they exhibit a large geographical distribution and range throughout various biomes (Da Fonseca et al. 1996), which, according to Krasnov et al. (2003), are among the most promising factors for the discovery of a new species of flea. In fact, a new species of *Tunga* and located in the carapace of *Zaedyus pichiy* and perforating the osteoderms is being now described by Ezquiaga et al. (unpublished work) from Argentina. Additionally, it is important to verify whether, in fact, Artiodactyla species represent true hosts for *Tunga* species because Rodrigues and Daemon (unpublished work) also found *T. trimamillata* on the capybara *H. hydrochaeris* in Brazil. In contrast, thus far, only *M. americana* of the 54 Brazilian species of Didelphimorphia (Reis et al. 2006) has been found to be infested with neosomes (Bossi 2003).

However, in spite of these possibilities, certain challenges exist for obtaining neosomes: (i) the extraction of the ectoparasites of mammals without killing the host in studies that do not allow the death of the host; (ii) incomplete inspection of the entire body of the animal by the collector, with the primary sites of attachment such as the belly, paws, ears, and tails not being examined; (iii) misdiagnosis, with the lesions being confused with larvae of *Cuterebra*, cicatrization of animals’ bites (Beaucournu et al. 2012b) or abscesses, mycosis, larva migrans, and other symptoms; and (iv) misidentifications with other species of *Tunga* because the taxonomic identifications are not always performed by experts. In fact, some occurrences attributed to *T. penetrans* on certain hosts are likely incorrect (Linardi et al. 2013).

In fleas, only adults are ectoparasitic, whereas the immature stages develop in the soil, inside or close to the nests of the respective hosts and, consequently, are susceptible to predation or environmental modifications. The prevalence and mean abundance of these ectoparasites could thus assist nature conservation studies because they can serve as indicators of eventual alterations when compared temporally in a single locality. For *T. penetrans*, the natural habitat is primarily the sandy and warm soil of deserts and beaches (Veraldi and Schianchi 1999), thus justifying the vernacular names sand flea, sandflöh, and puce de sable, as cited by Beaucournu et al. (2012b) and Cunha (1914). Granular soils, which are considered favorable for *T. penetrans* by the majority of authors, are also favorable for other congeneric species (Beaucournu et al. 2012a). However, both adults and immature young of other species of *Tunga* must be searched for in the nests and burrows of their respective hosts, especially those of edentates and rodents. On tropical beaches, the dispersion of *T. penetrans* is ensured by infested dogs. Additionally, horse manure used to fertilize soils aids in sand flea dissemination because both *T. penetrans* and *T. trimamillata* also live in stables and stock farms as well as in the soil and dust close to farms. Other fecal matter such as bat guano supports the development of *H. pulex* (Hastriter and Méndez 2000; Tipton and Méndez 1966).

It seems that the soil temperature, air temperature, and air humidity do not markedly affect the presence of *T. penetrans* in soil samples, as observed by Linardi et al. (2010) in three geographical regions of Brazil: Fortaleza (Ceará State), Barra do Garças (Mato Grosso State), and Alto Alegre (Roraima State). Larvae of this species were found in the sand in a depth of 2–5 cm (Nagy et al. 2007). Certain species are univoltine, as they are collected only during one season, including *T. caecigena* during the cold season and *T. callida* during winter months in China (Jordan 1962); *T. monositus* in Utah from October through April (Hastriter 1997); *T. libis* in June (Smit 1962), October, and November in Chile (Smit 1968); *T. bonneti* from July to December in Chile (Beaucournu et al. 2012a); and *T. hexalobulata* during the dry-cool season from April to September in Brazil (De Avelar et al. 2013). Similarly, both *T. penetrans* and *T. trimamillata* develop better in the dry season (Pampiglione et al. 2009), thus confirming the findings of Hoeppli (1963), who reported for *T. penetrans* in Africa that “the number of sandfleas greatly decreases during the rainy season.” With respect to the species of *Tunga* found on edentates, data included as material deposited in the Rothschild collection of fleas of the British Museum (Hopkins and Rothschild 1953) indicate November as the month in which *T. travassosi*, *T. terasma*, and *T. bondari* were collected, which corresponds to the rainy season in Brazil. For *T. caecata*, Meira (1934) observed neosomes on a rat’s ear in the months of December, February, and April, with only the latter included in the dry-cool season in São Paulo, Brazil.

Concerning other geographical factors, only the latitude, with respect to its influence on temperature, would play a role in distribution because the same species can be collected at different altitudes (Beaucournu et al. 2012a). In Chile, *T. bonneti* occurs more frequently from July to December, decreasing in January and February (Beaucournu et al. 2012a).

Although the treatment and prophylaxis of tungiasis are not within the scope of this review, it is interesting to note that these actions have only been performed in humans and domestic animals when parasitized. Treatment consists of local excision or sterile curettage. Parasitized dogs can be treated with a subcutaneous application of ivermectin and healing ointments and repellent (Corrêa et al. 2012).

In humans, prophylactic measures include the wearing of shoes, improving hygiene, use of gloves for handling manure, sweeping floors, and the restriction of free movement of people and infested animals among households. According to Matias (1989), in some municipalities of Rio Grande do Sul State, Brazil, trucks carrying sand and sheaves of grass used for construction of houses can also act in the dissemination of this species.

Tetanus vaccination is recommended to prevent secondary infection. Both within and around the residences in two municipalities of Rio Grande do Sul State, Brazil, the pyrethroids cypermethrin and deltamethrin were used as a measure of environmental control (Matias 1991). Given the diversity of habitats, Linardi (1998) claimed that integrated actions were required for the control of tungiasis, involving clinicians, veterinarians, biologists, public health specialists, and technicians and companies responsible for vector control. An overview of human tungiasis and including risk factors, treatment, repellents, prevention, control, and healthcare stakeholders has been presented by Karunamoorthi (2013).

Until recently, tungiasis provoked by *T. penetrans* had been reported in approximately 70 nations, especially in Latin America and Sub-Saharan Africa, most commonly affecting poor populations (Pampiglione et al. 2009). The number of cases has been increasing, as reported in several publications worldwide. Now, tungiasis is endemic or potentially endemic to 89 countries with varying degrees of incidence and prevalence, which vary in relation to the area and population studied. It has been estimated that the prevalence of tungiasis may reach more than 50 % of the population in some of the hyperendemic zones, with often recurrent and sometimes massive parasitic infestations that are responsible for superinfections (Karunamoorthi 2013).

In Brazil alone, more than 10^6^ individuals are at risk for severe tungiasis (Heukelbach et al. 2001). In the villages of Tanzania, jigger parasitization is locally referred as *inzyogo*, which means “the disease of the dirty people” (Mazigo et al. 2012). The treatment involves surgical extraction of the sand flea under sterile conditions, but due to the low socioeconomic status of some villages in Tanzania and Kenya, pins and other unsterilized equipment are being shared for jigger removal, leading to the possible further spread of HIV and AIDS and other diseases and increased opportunity for bacterial infection through the new open wounds that are often left uncovered (Wachira 2012).

In recent decades, ecotourism in forest areas, caves, and mountains has become another cause of the increase in the chances of human infestation by sand fleas, which usually parasitize other animals. Additionally, as a result of the expansion of urban areas into wild environments, the opportunities for contact between domestic and wild animals have increased, thus facilitating the exchange of ectoparasites. For this reason, it is possible that sand fleas parasitizing wild mammals may be found on domestic mammals and vice versa. In fact, this situation may be the case for *T. penetrans* and *T. trimamillata*, as pasturelands for cattle are now found in cerrado areas occupied by rodents and edentates.

## Electronic supplementary material

Below is the link to the electronic supplementary material.

ESM 1 (PDF 1855 kb)

ESM 2 (PDF 466 kb)

## References

[CR1] Antunes JMAP, Demoner LC, Martins IVF, Zanine MS, Deps DP, Pujol-Luz JR (2006). Registro de *Dasypus novemcinctus* (Mammalia: Xenarthra) em Alegre, Estado do Espírito Santo, Brasil. Rev Bras Parasitol Vet.

[CR2] Ariza L, Seidenschwang M, Buckendahl J, Gomide M, Feldmeier H, Heukelbach J (2007). Tungíase: doença negligenciada causando patologia grave em uma favela de Fortaleza, Ceará. Rev Soc Bras Med Trop.

[CR3] Audy JR, Radovsky FJ, Vercammen-Grandjean PH (1972). Neosomy: radical intrastadial metamorphosis associated with arthropod symbioses. J Med Entomol.

[CR4] Barnes AM, Radovsky FJ (1969). A new *Tunga* (Siphonaptera) from the nearctic region with description of all stages. J Med Entomol.

[CR5] Beaucournu J-C, Mergey T, Muñoz-Leal S, González-Acuña D (2012). Description de *Tunga bonneti* n. sp. du Chili (Siphonaptera: Tungidae) et notes sur su spécificité, sa chorologie, son dermecos et sa phénologie. Parasite.

[CR6] Beaucournu J-C, Degeilh B, Mergey T, Muñoz-Leal S, González-Acuña D (2012). Le genre *Tunga* Jarocki, 1838 (Siphonaptera: Tungidae). I – Taxonomie, phylogénie, écologie, role pathogéne. Parasite.

[CR7] Bell A, Neely CL, Peeples J (1979). Tungiasis in Tennessee. South Med J.

[CR8] Bezerra SM (1994). Tungiasis: an unusual case of severe infestation. Int J Derm.

[CR9] Blanchard R (1890). Traité de Zoologie Médicale.

[CR10] Bossi DEP (2003) Associações entre artrópodes e pequenos mamíferos silvestres de três áreas serranas do Sudeste brasileiro. PhD thesis, Estadual University of Campinas, Campinas, Brasil

[CR11] Brothers W, Heckmann R (1975). Tungiasis (*Tunga penetrans*) in Utah. J Parasitol.

[CR12] Brumpt E (1949). Précis de Parasitologie.

[CR13] Cardoso A (1981). Generalized tungiasis treated with thiabendazole. Arch Dermatol.

[CR14] Carvalho RW, Almeida AB, Barbosa-Silva, Amorim M, Ribeiro PC, Serra-Freire NM (2003). The patterns of tungiasis in Araruama township, state of Rio de Janeiro, Brazil. Mem Inst Oswaldo Cruz.

[CR15] Clyti E, Coupple F, Deligny C, Jouary T, Sainte-Marie D, Pradinaud R (2003). Efficacité de la vaseline salycitée à 20 % dans le traitment dês tungoses profuses. À propôs de huit observations en Guyane Française. Bull Soc Path Exot.

[CR16] Cooper JE (1976). *Tunga penetrans* infestation in pigs. Vet Rec.

[CR17] Corrêa RS, Barros SRA, da Hora AS, Mota MRS, Silva Filho LE, da Silva NM (2012). Tungíase em população canina: caso na comunidade São João do Tupé, Manaus, Amazonas. Amazon Sci.

[CR18] Cunha RA (1914) Contribuição para o estudo dos sifonápteros do Brasil. Tese inaugural, Instituto Oswaldo Cruz, Rio de Janeiro, Brasil

[CR19] Da Fonseca GAB, Herrmann G, Leite YLR, Mittermeier RA, Rylands AB, Patton JL (1996). Lista anotada dos mamíferos do Brasil. Occ Pap Cons Biol.

[CR20] Da Silva LAF, Santana AP, Borges GT, Linhares GFC, Fioravanti MCS, Rabelo RE (2001). Aspectos epidemiológicos e tratamento da tungíase bovina no município de Jataí, estado de Goiás. Cienc Anim Brasil.

[CR21] De Avelar DM (2010) Sistemática e análise cladística das espécies neotropicais do gênero *Tunga* Jarocki, 1838 (Siphonapera: Tungidae). PhD thesis, Federal University of Minas Gerais, Belo Horizonte, Brasil

[CR22] De Avelar DM, Linardi PM (2010) Use of multiple displacement amplification as pre-polymerase chain reaction (pre-PCR) to amplify genomic DNA of siphonapterids preserved for long periods in scientific collections. Parasites & Vectors 3:86–91 10.1186/1756-3305-3-86 PMC2945329 20840790

[CR23] De Avelar DM, Linhares AX, Linardi PM (2012). A new species of *Tunga* (Siphonaptera: Tungidae) from Brazil with a key to the adult species and neosomes. J Med Entomol.

[CR24] De Avelar DM, Facury Filho EJ, Linardi PM (2013). A new species of *Tunga* (Siphonaptera: Tungidae) parasiting cattle from Brazil. J Med Entomol.

[CR25] De Moraes LB, Bossi DEP, Linhares AX (2003). Siphonaptera of wild rodents and marsupials trapped in three mountain ranges of the Atlantic Forest in Southeastern Brazil. Mem Inst Oswaldo Cruz.

[CR26] Eisele MJ, Heukelbach J, Van Mardk E, Melhorn H, Meckes O, Franck S, Feldmeier H (2003). Investigations on the biology, epidemiology, pathology and control of *Tunga penetrans* in Brazil. I. Natural history of tungiasis in man. Parasitol Res.

[CR27] Esbérard C (2001). Infestation of *Rhynchopsyllus pulex* (Siphonaptera: Tungidae) on *Molossus molossus* (Chiroptera) in Southeastern Brazil. Mem Inst Oswaldo Cruz.

[CR28] Ewing ME, Fox I (1943). The fleas of North America. Miscell Publ U S Dept Agric.

[CR29] Ezquiaga MC, Lareschi M, Aba AM, Navone GT (2008). Nuevos registros de pulgas (Siphonaptera) parasitas de dasipódidos (Mammalia: Xenarthra) em el noreste de la provincia de Buenos Aires, Argentina. Mastozool Neotr.

[CR30] Feldmeier H, Heukelbach J, Eisele M, Sousa AQ, Barbosa LM, Carvalho CBM (2002). Bacterial superinfection in human tungiasis. Trop Med Int Hlth.

[CR31] Feldmeier H, Eisele M, Van Marck E, Mehlhorn H, Ribeiro R, Heukelbach J (2004). Investigations on the biology, epidemiology, pathology and control of *Tunga penetrans* in Brazil. IV. Clinical and histopathology. Parasitol Res.

[CR32] Feldmeier H, Witt L, Schwalfenberg S, Linardi PM, Ribeiro RA, Capaz RAC, Marck EV, Meckes O, Mehlhorn H, Mencke N, Heukelbach J (2007). Investigations on the biology, epidemiology, pathology and control of *Tunga penetrans* in Brazil. VI. Natural history of the infestation in laboratory-raised Wistar rats. Am J Trop Med Hyg.

[CR33] Fioravanti ML, Gustinelli A, Onore G, Pampiglione S, Trentini M (2006). Presence of *Tunga trimamillata* (Insecta, Siphonaptera) in Peru. Parasite.

[CR34] Fischer P, Schmetz C, Bandi C, Bonow I, Mand S, Fischer K, Büttner DW (2002). *Tunga penetrans*: molecular identification of *Wolbachia* endobacteria and their recognition by antibodies against proteins of endobacteria from filarial parasites. Exp Parasitol.

[CR35] Fitzsimmons SWM (1966). Some helminth and arthropod parasites common to man and animals in Malawi. Ann Trop Med Parasit.

[CR36] Frank R, Melaun C, Martins MM, Santos ALQ, Heukelbach J, Klimpel S (2012). *Tunga penetrans* and further parasites in the giant anteater (*Myrmecophaga tridactyla*) from Minas Gerais, Brazil. Parasitol Res.

[CR37] Geigy R, Herbig A (1949). Die hypertrophie der Organe beim Weibchen von *Tunga penetrans*. Acta Trop.

[CR38] Goldman L (1976). Tungiasis in travelers from tropical Africa. J Amer Med Ass.

[CR39] Greco JB, Sacramento E, Tavares-Neto J (2001). Chronic ulcers and myasis as ports of entry for *Clostridium tetani*. Braz J Infect Dis.

[CR40] Hastriter MW (1997). Establishment of the tungid flea, *Tunga monositus* (Siphonaptera: Pulicidae), in the United States. Great Bas Nat.

[CR41] Hastriter MW, Méndez E (2000). A review of the flea genera *Hectopsylla* Frauenfeld and *Rhynchopsyllus* Haller (Siphonaptera: Pulicidae). Proc Entomol Soc Wash.

[CR42] Heukelbach J (2005). Tungiasis. Rev Inst Med Trop S Paulo.

[CR43] Heukelbach J (2006). Revision on tungiasis: treatment options and prevention. Exp Rev Anti-Inf Ther.

[CR44] Heukelbach J, Oliveira FAS, Hesse G, Feldmeier H (2001). Tungiasis: a neglected health problem of poor communities. Trop Med Int Health.

[CR45] Heukelbach J, Wilcke T, Eisele M, Feldmeier H (2002). Ectopic localization of tungiasis. Amer J Trop Med Hyg.

[CR46] Heukelbach J, Bonow I, Witt L, Feldmeier H, Fischer P (2004). High infection rate of *Wolbachia* endobacteria in the sand flea *Tunga penetrans* from Brazil. Acta Trop.

[CR47] Heukelbach J, Costa AML, Wilcke T, Mencke, Feldmeier H (2004). The animal reservoir of *Tunga penetrans* in severely affected communities of North-East Brazil. Med Vet Entomol.

[CR48] Heukelbach J, Jackson A, Ariza L, Calheiros CM, Soares VL, Feldmeier H (2007). Epidemiology and clinical aspects of tungiasis (sand flea infestation) in Alagoas State, Brazil. J Infect Dev Countries.

[CR49] Hoeppli R (1963). Early references to the occurrence of *Tunga penetrans* in tropical Africa. Acta Trop.

[CR50] Holland GP (1964). Evolution, classification, and host relationships of Siphonaptera. Ann Rev Entomol.

[CR51] Hopkins GHE, Rothschild M (1953). An illustrated catalogue of the Rothschild collection of fleas (Siphonaptera) in the British Museum (Natural History), vol 1. Tungidae and Pulicidae.

[CR52] Ioff IG (1950). The alakurt. Mater Pozn Faun Flor SSSR.

[CR53] Johnson PT (1957). A classification of Siphonaptera of South America with descriptions of new species. Mem Entomol Soc Wash.

[CR54] Jordan K (1962). Notes on *Tunga caecigena* (Siphonaptera: Tungidae). Bull Brit Mus (Nat Hist) Ent.

[CR55] Karunamoorthi K (2013). Tungiasis: a neglected epidermal parasitic skin disease of marginalized populations—a call for global science and policy. Parasitol Res.

[CR56] Kehr JD, Heukelbach J, Mehlhorn H, Feldmeier H (2007). Morbidity assessment in sand flea disease (tungiasis). Parasitol Res.

[CR57] Klimpel S, Mehlhorn H, Heukelbach J, Feldmeier H, Mencke (2005). Field trial of the efficacy of a combination of imidacloprid and permethrin against *Tunga penetrans* (sand flea, jigger flea) in dogs in Brazil. Parasitol Res.

[CR58] Krasnov BR (2008). Functional and evolutionary ecology of fleas. A model for ecological parasitology.

[CR59] Krasnov BR, Shenbrot GI, Mouillot D, Khokhlova IS, Poulin R (2003). What are the factors determining the probability of discovering a flea species (Siphonaptera)?. Parasitol Res.

[CR60] Lavoipierre MMJ, Radovsky F, Budwiser PD (1979). The feeding process of a tungid flea, *Tunga monositus* (Siphonaptera: Tungidae), and its relationship to the host inflammatory and repair response. J Med Entomol.

[CR61] Lewis RE (1998). Résumé of the Siphonaptera (Insecta) of the world. J Med Entomol.

[CR62] Li K-C, Chin T-H (1957). *Tunga callida* sp. nov., a new species of sandflea from Yunnan. Acta Entomol Sin.

[CR63] Lima AC (1943) Suctoria. In: Lima AC (ed) Insetos do Brasil, 4. Tomo, Escola Nacional de Agronomia, Rio de Janeiro, pp 17–71

[CR64] Lima AC, Hathaway CR (1946) Pulgas. Bibliografia, catálogo e hospedadores. Instituto Oswaldo Cruz, Rio de Janeiro

[CR65] Linardi P (1998). Tungíase: uma pulga diferente que provoca um problema persistente. Vet Pragas.

[CR66] Linardi PM, Guimarães LR (2000). Sifonápteros do Brasil.

[CR67] Linardi PM, Krasnov B (2013). Patterns of diversity and abundance of fleas and mites in the Neotropics: host-related, parasite-related and environment-related factors. Med Vet Entomol.

[CR68] Linardi PM, Calheiros CMC, Campelo-Junior EB, Duarte EM, Heukelbach J, Feldmeier H (2010). Occurrence of the off-host life stages of *Tunga penetrans* (Siphonaptera) in various environments in Brazil. Ann Trop Med Parasit.

[CR69] Linardi PM, De Avelar DM, Facury Filho EJ (2013). Establishment of *Tunga trimamillata* (Siphonaptera: Tungidae) in Brazil. Parasitol Res.

[CR70] Luchetti A, Mantovani B, Fioravanti ML, Trentini M (2004). *Wolbachia* infection in the newly described Ecaudorian sand flea *Tunga trimamillata*. Exp Parasitol.

[CR71] Luchetti A, Mantovani B, Pampiglione S, Trentini M (2005). Molecular characterization of *Tunga trimamillata* and *T. penetrans* (Insecta, Siphonaptera, Tungidae): taxonomy and genetic variability. Parasite.

[CR72] Luchetti A, Mantovani ML, Trentini M (2005). *Wolbachia* superinfection in an Ecuadorian sample of sand flea *Tunga penetrans*. Bull Infect.

[CR73] Luz JL, Costa LM, Gomes LAC, Esbérard CEL (2009). The chiggerflea *Hectopsylla pulex* (Siphonaptera: Tungidae) as an ectoparasite of free-tailed bats (Chiroptera: Molossidae). Mem Inst Oswaldo Cruz.

[CR74] Macchiavello A (1948). Siphonaptera de la costa sur-occidental de América (Primera lista y distribución zoo-geográfica). Bol Of Sanit Panamer.

[CR75] Maco V, Tantaleán M, Gotuzzo E (2011). Evidence of tungiasis in pre-Hispanic America. Emerg Inf Dis.

[CR76] Marshall AG (1981). The ecology of ectoparasitic insects.

[CR77] Mascarenhas RSC (2002) A co-evolução de Tunginae (Siphonaptera Pulicidae) e os Edentata (Mammalia). PhD thesis, Universidade Estadual Paulista, Botucatu, Brasil

[CR78] Matias RS (1989). Epidemia de tungíase no Rio Grande do Sul. Rev Soc Bras Med Trop.

[CR79] Matias RS (1991). Verificação da eficácia de diferentes inseticidas no controle ambiental de *Tunga penetrans* (L., 1758). Rev Soc Bras Med Trop.

[CR80] Mazigo HD, Bahemana E, Konje ET, Dyegura O, Mnyone LL, Kweka EJ, Kidenya BR, Heukelbach J (2012). Jigger flea infestation (tungiasis) in rural western Tanzania: high prevalence and severe morbidity. Trans R Soc Trop Med Hyg.

[CR81] Medri IM (2008) Ecologia e história natural do tatu-peba, *Euphractus sexcinctus* (Linnaeus, 1758), no Pantanal da Nhecolândia, Mato Grosso do Sul. PhD thesis, University of Brasília, Brasília, Brasil

[CR82] Meira JA (1934). Contribuição parasitológica para a epidemiologia da peste bubônica na cidade de São Paulo. Sobre as pulgas de rato da mesma cidade. Ann Paul Med Cir.

[CR83] Mittermeier RA, Myers N, Thomsen JB, Da Fonseca GAB, Olivieri S (1998). Biodiversity hotspots and major tropical wilderness areas: approaches to setting conservation priorities. Conserv Biol.

[CR84] Moraes FR, Hatayde MR, Costa AJ, Moraes JRE, Rocha UF (1992). Novo surto de *Tunga penetrans* (L., 1758) em bovinos: aspectos clínicos, anatomopatológicos e tratamento. Ars Vet.

[CR85] Müller P (1972). Centres of dispersal and evolution in the Neotropical region. Stud Neotrop Fauna Environ.

[CR86] Myers N, Mittermeier RA, Mittermeier CG, Da Fonseca GAB, Kent J (2000). Biodiversity hotspots for conservation priorities. Nature.

[CR87] Nagy N, Abari E, D’Haese J, Calheiros C, Heukelbach J, Mencke N, Feldmeier H, Mehlhorn H (2007). Investigations on the life cycle and morphology of *Tunga penetrans* in Brazil. Parasitol Res.

[CR88] Obengui P (1989). La tungose et le tétanos au C.H.U. de Brazzaville. Dakar Med.

[CR89] Pampiglione S, Trentini M, Gentili FM, Mendes JLX, Pampiglione C, Rivasi F (1998). *Tunga penetrans* (Insecta: Siphonaptera) in pigs in São Tomé (Equatorial Africa): epidemiological, clinical, morphological and histopathological aspects. Rev Elev Med Vet Pays Trop.

[CR90] Pampiglione S, Fioravanti ML, Gustinelli A, Onore G, Mantovani B, Luchetti A, Trentini M (2009). Sand flea (*Tunga* spp.) infections in humans and domestic animals: state of the art. Med Vet Entomol.

[CR91] Pedroso-de-Paiva D, Sobestiansky J, Dalla Costa OA, Varaschin D (1997). Aspectos epidemiológicos de um foco de tungíase (*Tunga penetrans*, Siphonaptera) em um sistema intensivo de suínos criados ao ar livre. Anais Esc Agron Vet.

[CR92] Pilgrim RLC (1993). An instance of tungiasis in New Zealand. New Zeal Med Journ.

[CR93] Pinto C (1930). Arthrópodes parasites e transmissores de doenças.

[CR94] Pinto C, Dreyfus A (1927). *Tunga travassosi* n. sp., parasita de *Tatusia novemcinctus* do Brasil. Bol Biol.

[CR95] Pollitzer R (1954) Plague. WHO, Geneve Reis NR, Peracchi A, Pedro WA, Lima IP (2006) Mamíferos do Brasil. Universidade Estadual de Londrina, Londrina

[CR96] Reis NR, Peracchi A, Pedro WA, Lima IP (2006) Mamíferos do Brasil. Universidade Estadual de Londrina, Londrina, Brasil

[CR97] Reiss F (1966). Tungiasis in New York City. Arch Derm.

[CR98] Ribeiro H (1974). Sifónapteros de Angola (Insecta, Siphonaptera). Estudo sistemático e dados bioecológicos interessando à epidemiologia da peste.

[CR99] Ribeiro JCVC, Coelho SC, Ruas JRM, Lana AMQ, Carvalho AU, Facury Filho EJ, Saturnino HM, Reis RB (2007). Infestação de *Tunga penetrans* Siphonaptera: Tungidae em cascos de vacas leiteiras F1 Holandês-Zebu. Arq Bras Med Vet Zootec.

[CR100] Rodrigues DF, Daemon E, Rodrigues FSF (2008). Caracterização da população de ectoparasitos em cães de núclos de espansão urbana em Juiz de Fora, Minas Gerais, Brasil. Rev Bras Parasitol Vet.

[CR101] Rothschild M (1992). Neosomy in fleas, and the sessile life-style. J Zool Lon.

[CR102] Ruthe H (1961). Fussleiden der Elefanten. Wissenschaftliche Zeitschrift der Humboldt-Universität zu Berlin. Mathematisch-Naturwissenschaftliche Reihe.

[CR103] Sakaguti K, Jameson EW (1962). The Siphonaptera of Japan. Pacific Insects Monogr.

[CR104] Sanusi ID, Brown EB, Shepard TG, Grafton WD (1989). Tungiasis: report of one case and review of the 14 reported cases in the United States. J Amer Acad Derm.

[CR105] Smit FGAM (1962). A new sand-flea from Ecuador. Entomologist.

[CR106] Smit FGAM (1968). Siphonaptera taken from formalin-traps in Chile. Zool Anz.

[CR107] Soria MF, Capri JJ (1953). Tetanos y “piques”. Prensa Med Argent.

[CR108] Stanko M, Miklisova D, Goüy de Bellocq J, Morand S (2002). Mammal density and patterns of ectoparasite species richness and abundance. Oecologia.

[CR109] Tipton VJ, Méndez E, Wenzell RL, Tipton VJ (1966). The fleas (Siphonaptera) of Panama. Ectoparasites of Panama.

[CR110] Tonge BL (1989). Tetanus from chigger flea sores. J Trop Pediat.

[CR111] Traub R (1972). The Gunong Benom Expedition 1967. XII. Notes on zoogeography, convergent evolution and taxonomy of fleas (Siphonaptera), based on collection from Gunong Benom and elsewhere in South-East Asia. 2. Convergent evolution. Bull Brit Mus Nat Hist Zool.

[CR112] Traub R, Starcke H, Traub R (1980). The zoogeography and evolution of some fleas, lice and mammals. Fleas.

[CR113] Traub R, Kim KC (1985). Coevolution of fleas and mammals. Coevolution of parasitic arthropods and mammals.

[CR114] Ugbomoiko S, Ariza L, Heukelbach J (2008). Pigs are the most important animal reservoir for *Tunga penetrans* (jigger flea) in rural Nigeria. Trop Doct.

[CR115] Vaz Z, Rocha UF (1946). *Tunga penetrans* (L., 1758), “bicho de pé” em gado bovino. Livro de homenagem a RF Almeida.

[CR116] Veraldi S, Schianchi R (1999). Tungiasis. Eur J Dermatol.

[CR117] Veraldi S, Valsecchi M (2007). Imported tungiasis: a report of 19 cases and review of the literature. Int J Dermatol.

[CR118] Veraldi S, Camozzi S, Scarabelli G (1996). Tungiasis presenting with sterile pustular lesions on the hand. Acta Derm-venereol Stockh.

[CR119] Verhulst A (1976). *Tunga penetrans* (*Sarcopsylla penetrans*) as a cause of agalactia in sows in the Republic of Zaire. Vet Rec.

[CR120] Vernalha MM, Gabardo JC, Da Silva RP, Macedo Junior S (1984). Ocorrência da *Tunga penetrans* (L., 1758) – (Suctoria - Tungidae) no município de Curitiba – estado do Paraná – Brasil. Acta Biol Par.

[CR121] Wachira A. Infestation of the jigger flea in resource-poor communities in Africa. Consultance Africa Intelligence. www.consultancyafrica.com. Accessed 02 May 2012

[CR122] Wagner J (1932). *Tunga bondari*, eine neue art der sandflöhe. Novit Zool.

[CR123] Wenzell RL, Tipton VJ (1966). Ectoparasites of Panama.

[CR124] Whiting MF, Whiting AS, Hastriter MW, Ditmar KA (2008). A molecular phylogeny of fleas (Insecta: Siphonaptera): origins and host associations. Cladistics.

[CR125] Widmer CE, Azevedo FCC (2012). Tungiasis in a free-ranging jaguar (*Panthera onca*) population in Brazil. Parasitol Res.

[CR126] Wilson DE, Reeder DM (2005). Mammal species of the world: a taxonomic and geographic reference.

[CR127] Witt LH, Linardi PM, Meckes O, Schwalfenberg S, Ribeiro RA, Feldmeier H, Heukelbach J (2004). Blood-feeding of *Tunga penetrans* males. Med Vet Entomol.

[CR128] Wolffhügel K (1910). Die Flöhe (Siphonaptera) der Haustiere. Z Infekt Krank Haustiere.

[CR129] Wu K (1930). A study of the common rat and its parasites. Ling Sci J.

[CR130] Yang HS (1955). Notes on the sandflea, *Tunga caecigena* Jordan & Rothschild in Foochow. Acta Ent Sinica.

[CR131] Yulong W, Jiji LM, Lemons DE, Weinbaumet S (1995). A non-uniform three-dimensional perfusion model of rat tail heat transfer. Phys Med Biol.

