# Supplementary material for: Neosomes of tungid fleas on wild and domestic animals
Source: Parasitol Res. 2014 Aug 21;113(10):3517–33. doi: 10.1007/s00436-014-4081-8 (PMC4172993; doi:10.1007/s00436-014-4081-8)
Supplement: Supplementary file 2 — (PDF 466 kb) [file 436_2014_4081_MOESM2_ESM.pdf]

https://mail.google.com/mail/u/0/#inbox/145d876 | Parasitology Research - Springer | Editorial Manager® | Enc: RE: reproduction of fig... | Google Tradutor

Google | +Daniel | Share

Gmail | More | 1 of 10,616

COMPOSE

Inbox (3,048)

Important

Chats

Sent Mail

Search people...

Michel Valim
Ana Nilce Elkhoury
Ana Paula Salles...
Andreza Pain Mar...
Atatília Ostini
Camilla Ribeiro
Cândido Júnior
claudia mendonça
Erick Johnson Ca...
Igor Castro
Marcelo Biólogo
Matheus TCN!!! C...
michaelle ohana

----- Mensagem encaminhada -----
De: Alan Kahan <AKahan@entsoc.org>
Para: Pedro Marcos Linardi <linardi@icb.ufmg.br>
Enviadas: Thu, 08 May 2014 14:58:23 -0300 (BRT)

Assunto: RE: reproduction of figures

Yes, permission is granted.

Alan Kahan

Alan Kahan / Director of Publications & Communications

Entomological Society of America / 3 Park Place, Suite 307 / Annapolis, MD 21401-3722 / USA

Phone: +1 301-731-4535, ext. 3020 / Direct Dial: +1 240-696-3749

Fax: +1 301-731-4538 / Email: akahan@entsoc.org

From: Pedro Marcos Linardi [mailto:linardi@icb.ufmg.br]

Sent: Thursday, May 08, 2014 9:36 AM
To: Alan Kahan
Subject: RE: reproduction of figures

Dear Dr. Kahan,

Thanks for your prompt reply. However, I'm in need of one more photo also published in Journal Medical Entomology, concerning the neosome of
Tunga monositus:
Lavoipierre et al.1979: The feeding process of a tungid flea.... Vol. 15, no.3, page 189, fig.3.
May I have permission to reuse it?
Sincerly,
Prof. Pedro M. Linardi

IBR - Ar Mandado

linardi@icb.ufmg.br

Add to circles

Show details

Ad

Campeões do Carinho
A votação dos Campeões de Carinho está terminando. Acesse e vote!
careinspirescare.com/br/campeoes

PT 11:55 02/06/2014

https://mail.google.com/mail/u/0/#inbox/145d876 | Parasitology Research - Springer | Editorial Manager® | Enc: RE: reproduction of fig... | Google Tradutor

Google | +Daniel | Share

Gmail | More | 1 of 10,616

COMPOSE
Inbox (3,048)
Important
Chats
Sent Mail
Search people...
Michel Valim
Ana Nilce Elkhoury
Ana Paula Salles...
Andreza Pain Mar...
Atatília Ostini
Camilla Ribeiro
Cândido Júnior
claudia mendonça
Erick Johnson Ca...
Igor Castro
Marcelo Biólogo
Matheus TCN!!! C...
michaelle ohana

----- Mensagem encaminhada -----
De: Alan Kahan <AKahan@entsoc.org>
Para: Pedro Marcos Linardi <linardi@icb.ufmg.br>
Enviadas: Tue, 06 May 2014 15:29:37 -0300 (BRT)
Assunto: RE: reproduction of figures

Prof. Pedro M. Linardi
Departamento de Parasitologia
Instituto de Ciências Biológicas
Universidade Federal de Minas Gerais
Caixa Postal 486
31.270-901 Belo Horizonte, Minas Gerais
Brasil

Dear Prof. Linardi,

The Entomological Society of America grants you permission to reuse the figures cited below as part of a review article you are writing for another journal.

De Avelar, Daniel M.; Linhares, Arício X.; Linardi, Pedro M. 2012. A New Species of *Tunga* (Siphonaptera: Tungidae) From Brazil With a Key to the Adult Species and Neosomes. Journal of Medical Entomology, 49(1): 23-28, Figure 1

De Avelar, Daniel M.; Facury Filho, Elias J.; Linardi, Pedro M. 2013. A New Species of *Tunga* (Siphonaptera: Tungidae) Parasitizing Cattle From Brazil. Journal of Medical Entomology, 50(4): 679-684, Figure 1.

Sincerely,
Alan Kahan

Alan Kahan / Director of Publications & Communications

Entomological Society of America / 3 Park Place, Suite 307 / Annapolis, MD 21401-3722 / USA

Phone: +1 301-731-4535, ext. 3020 / Direct Dial: +1 240-696-3749

[illegible]

IBR - Ar Mandado
linardi@icb.ufmg.br
Add to circles
Show details
Ad
Campeões do Carinho
A votação dos Campeões de Carinho está terminando. Acesse e vote!
careinspirescare.com/br/campeoes

PT 11:54 02/06/2014

From:
Pedro Marcos Linardi [mailto:linardi@icb.ufmg.br]
Sent: Thursday, May 08, 2014 4:01 PM
To: justine@mnhn.fr
Subject: reproduction of figures

Dear Dr.
Justine,

I'm
completing a review article about neosomes of *Tunga* (Siphonaptera)
which should be submitted for publication in another
journal. To do so, I need your permission for
reproduction of some figures of species described and
that has been included in the Parasite, respectively:
Beaucournu et
al. 2012: Description de *Tunga bonneti* n.
sp.... Figs 2, 4 and 5, page 212, vol.19. I also would
like to reuse the figs 3A and 3B, pag. 13, vol. 10,
2003, concerning T. penetrans and published by
Pampiglione et al.
I inform you that I am writing
along with Dr. Beaucournu and others the second part
of the article "Le genre *Tunga*" that will be
submitted in due course to the Parasite for
publication.
Sincerely,
Prof. Pedro M. Linardi
Departamento de Parasitologia ICB
Universidade Federal de Minas
Gerais

https://mail.google.com/mail/u/0/#inbox/1465cfd | Parasitology Research - Springer | Editorial Manager® | Enc: Re: FW: reproduction o... | Google Tradutor

Gmail

Inbox (3,048)
Important
Chats

2 of 10,616

----- Mensagem encaminhada -----
De: Zala Ngita <zala.ngita@edpsciences.org>
Para: linardi@icb.ufmg.br
Cc: Jean-Lou JUSTINE <justine@mnhn.fr>
Enviadas: Mon, 12 May 2014 12:01:50 -0300 (BRT)
Assunto: Re: FW: reproduction of figures

Dear Prof. Pedro M. Linardi,

The Creative Commons license allows it, you can reproduce the figures

of species described in these articles, however, you must cite the source.

Best regards,

Zala NGITA

EDP Sciences

Parasite: http://www.parasite-journal.org

From:
Pedro Marcos Linardi [mailto:linardi@icb.ufmg.br]
Sent: Thursday, May 08, 2014 4:01 PM
To: justine@mnhn.fr
Subject: reproduction of figures

Plano de Saúde - ADEP

IBR - Ar Mandado
Linha de ar, Ar mandado, Mascaras Cavalete Filtrante, Engate Rapido.
www.ibrbrasil.ind.br

11:56
02/06/2014
